# Chiari malformation type I and basilar invagination originating from atlantoaxial instability: a literature review and critical analysis

**DOI:** 10.1007/s00701-020-04557-6

**Published:** 2020-09-07

**Authors:** Bernhard Meyer, Arthur Wagner, Lukas Grassner, Nikolaus Kögl, Sebastian Hartmann, Claudius Thomé, Maria Wostrack

**Affiliations:** 1 Department of Neurosurgery, Klinikum rechts der Isar, Technical University of Munich School of Medicine, Ismaninger Str. 22, 81675 Munich, Germany; 2 Department of Neurosurgery, Medical University Innsbruck, Innsbruck, Austria

## Response to “The Battle Against An Invisible Enemy”

It seems hard to formulate an appropriate response to the letter written by Tripathi and Mohindra, in which they commented on our review.

We have simply stated in our review that we do not see enough evidence after reviewing the relevant literature to change the principle concept of treating CM type 1 patients.

The answer to this in their letter is in a nutshell, yes there is because Prof. Goel says so.

I am sorry to say that this answer is not acceptable in the year 2020 to the majority of peers.

If you want to convince the community, you have to execute a comparative prospective multicentric study with predefined outcome parameters, which is independently controlled. No less no more.

The notion that this technique works only in one department and everybody should go there to learn it or leave it is simply not acceptable.

CVJ pathologies are too diverse and complex according to me and the vast majority of peers, and a single form of treatment is unlikely to be the solution for all.

However, I am happy to be convinced of the opposite by a study that fulfills the general criteria outlined above.

